# Familian Mediterranean Fever: genetic characterization in Georgian population

**DOI:** 10.1186/1546-0096-12-S1-P264

**Published:** 2014-09-17

**Authors:** Maka Ioseliani, Maia Lekishvili, Nunu Shelia

**Affiliations:** 1 M.Iashvili Children's Central Hospital, Tbilisi, Georgia

## Introduction

FMF is the most common mendelian autoinflamatory syndrome, resulting from autosomal recessive mutations in the MEFV locus. This disorder occurs most frequently among Sephardic Jewish, Arab, Armenian andTurkish populations. FMF occurs at lower frequeccies in other Mediterranean populations and ethnicities.

## Objectives

In Georgia this disorder was detected mainly in ethnic Jewish and Armenians. We present cases of FMF in ethnic Georgians, that we have diagnosed in our department from the day of its foundation (2007) up today (2014).

## Methods

We suspected FMF in 37 patients, the diagnosis was based on typical features. The FMF mutations were investigated in all patients. As a result FMF was proved in 37 cases is in investigation stage.

## Results

Of the 37 patients 19(52.8%) are females, 18(47.2%)are males and the age ranged from 2 to16. A positive family history of FMF was noted in 5(13.5%).Two patient has developed amyloidosis(mutationM694V/M694V). 27 of the patients had mutation M694V/M694V. 3 had mutation M680i/M694V. Another had M680I/M964V, M6801c/R761H, M680I/V726A, E148Q/M694V,M964V/WT. We have not colchicines resistant patient.

## Conclusion

Our study has approved that FMF occurs not only among Mediterranean population but among others including Georgians.In our population mostly frequent type of mutation is M694V/M694V.

## Disclosure of interest

None declared.

